# No evidence of inequality aversion in the investment game

**DOI:** 10.1371/journal.pone.0204392

**Published:** 2018-10-23

**Authors:** Ismael Rodriguez-Lara

**Affiliations:** Middlesex University London, Business School, Hendon Campus, The Burroughs, London NW4 4BT, United Kingdom; Groupe ESC Dijon Bourgogne, FRANCE

## Abstract

We report experimental evidence on second-movers’ behavior in the investment game (also known as the trust game) when there exists endowment heterogeneity. Using a within-subject analysis, we investigate whether or not second-movers exhibit some taste for inequality aversion by returning a larger (smaller) share of the available funds to first-movers who are initially endowed with a lesser (larger) endowment, respectively. Our data suggest that second-movers do not take into consideration the level of endowments when making their decisions as their behavior is consistent across distribution of endowments; i.e., they return the same proportion of the available funds regardless of the endowments. We indeed find that some second-movers have a tendency to return what they have received from first-movers. In our data, there is also a substantial proportion of second-movers who are selfish and return nothing. (JEL Codes: C72, C91, D3, D63).

## Introduction

Models of inequality aversion posit that individuals dislike payoff differences, thus they may be willing to forego monetary payoffs to reduce inequality (e.g., see [1], [2]). In the last decades, these models have emerged as a useful tool for reconciling observed behavior in laboratory experiments with the standard assumption that individuals care only about their own material payoffs. Although intuitively attractive and capable of predicting behavior in many different settings, inequality aversion may sometimes be in conflict with other behavioral motives such as reciprocity.

Take, for example, the investment game (also known as the trust game) in [3]. In this sequential game, the first-mover (hereafter, the investor, she) has to decide which part of her endowment (if any) she wants to send to a second-mover (hereafter, the allocator, he). Any amount sent is multiplied by three before the allocator decides how much of the generated surplus to give back. Assume that subjects differ in their initial endowments, if the unequal distribution of endowments is in favor of the investor then reciprocal behavior from the allocator may in fact induce more inequality. This type of situation is studied in [4], who show that allocators have a tendency not to pay back if doing so would increase inequality (see also [5]). Arguably, the allocator’s decision can also reduce payoff inequality when his endowment is larger than the investor’s. In that case, returning a large share of the available funds would lessen the difference in final payoffs. This may not necessarily be interpreted as reciprocal behavior but rather as a choice in pursuit of equality (i.e., allocators may dislike that their payoffs are ahead).

In this paper, we investigate how allocators reciprocate the investors’ decision when there is endowment heterogeneity. More specifically, we want to study whether (and how) allocators take into consideration differences in endowments when making their decision about the share of the available funds to return. Our aim is to study how subjects respond to changes in the context, thus we rely on a within-subject design. This design choice allows us to investigate also the willingness to reciprocate in a repeated context. Empirically, this is a relevant question as many economic situations require that agents interact repeatedly.

Our working conjecture is that allocators want to reduce the difference in final payoffs because they are inequality-averse. As a result, allocators will return a higher share when investors are at a relative disadvantage with regard to the initial distribution of endowments, compared with the case in which they are at a relative advantage. We test this predictions using the behavioral data in [6]. Our findings suggest that allocators make a division of the available funds that is consistent across the different distribution of endowments. On average, allocators do not tend to compensate investors who are in a position of disadvantage with regard to the initial level of endowments, nor do they tend to keep a larger fraction of the available funds when they are in a disadvantage position. Allocators seem to return what they have received without accounting for differences in the initial endowments.

In the light of this finding, we posit that reciprocity can be an alternative explanation to reconcile with our findings. Because the prediction for selfish agents is that allocators will return nothing, there is a definition of reciprocity that assumes that allocators are reciprocal whenever they return something positive (see [4]). Other models include the role of intentions; (see e.g., [7], [8], [9], [10], [11], [12], [13]). A key element in the intention-based models is that allocators reciprocate depending on their beliefs about the kindness of the investor; thus a particular amount received could be differently reciprocated depending on whether the investor’s endowment was low or high, or the efficiency factor that multiplies any amount sent by the investor to the allocator. We follow [14], [15], [16], [17], or [18], and refer to reciprocity as the allocator’s willingness to pay back what he has received from the investor. We find evidence that is partially consistent with this interpretation. There is also a substantial proportion of subjects (roughly 40%) that behave in accord with the idea of selfish preferences as they tend to keep what is generated after the investment decision.

Our paper is not the only paper that attempts to test inequality-aversion. Some evidence exists that subjects have a taste for egalitarian outcomes (e.g., [19], [20], [21], although the idea of inequality aversion is sometimes rejected (e.g., [22], [23]), especially when it is tested against other behavioral motives, such as efficiency ([24], [25]) or impure altruism ([26]). In the case of the investment game, some studies attempt to disentangle the different motives behind trust and reciprocity. These include the work of [10] or [27] who assess the role of intentions, and investigate the importance of altruism and expectations on behavior (see also [9], [11], [28]). To the best of our knowledge, however, there is no direct test for inequality aversion in the investment game when the endowment of the investor and the allocator are systematically varied across different rounds of the experiment. The closest paper to ours are [4] or [5], who compare allocators’ behavior in a symmetric situation (where investors and allocators receive the same endowment), with behavior in an asymmetric situation (where allocators are given a lesser endowment). Besides some noteworthy differences in experimental design (e.g., these papers employ a between-subject analysis thus no decision is ever repeated) we also make allocators play with a higher endowment, so that we can test whether they are more willing to pay back if their action reduces inequality.

Other experimental papers that investigate investors’ behavior in the presence of endowment heterogeneity include [29], [30], [31], [32], or [33]. These papers mainly focus on the effects of inequality on the investment decision (or the level of trust), thus the allocators’ behavior and their willingness to restore equality are often disregarded. In addition, all these papers induce inequality by paying subjects different show-up fees or endowments but these are kept constant across rounds, therefore they cannot assess how subjects response to changes in the level of endowments. This research question touches upon [34], who play a variant of the trust game with people from the British Household Panel Survey and find that a decrease in the respondent’s wealth (when comparing current and past income) reduces trustworthiness. Our contribution to this literature is to use a within-subject design to shed light into the allocators’ behavior in a repeated context when the level of endowments is varied across rounds.

## On the role of reciprocity and inequality-aversion in the investment game

Consider the investment game in [3], where the investor (subject *i*) and the allocator (subject *a*) are assumed to be endowed with *e*_*i*_ ≥ 0 and *e*_*a*_ ≥ 0, respectively. In the first stage of the game, the investor has to decide the amount X in [0, *e*_*i*_] that she wants to send to the allocator. The allocator receives 3*X* and has to decide the percentage *y* in [0, 1] of the available funds (3X) that he wants to return. The subjects’ final payoffs in the game are computed as follows:

$$\begin{array}{l} \pi^{i}(e_{i},X,y)≔e_{i}+X(3y-1)\geq 0\\ \pi^{a}(e_{a},X,y)≔e_{a}+3X(1-y)\geq 0 \end{array}$$

Transfers between the investor and the allocator have been usually interpreted as evidence in favor of trust and trustworthiness (see [35], [36], [37] for a review of the results). Our aim in this paper is to investigate the predictive power of inequality aversion in a within-subject design, in which subjects will face different partners across rounds.

When the investor and the allocator differ in their initial endowment, the allocator may want to equalize payoffs (see [4], [17]). To build upon this possibility, we consider that the allocator suffers a cost if his final payoff differ from the investor’s one (e.g., [1], [2]). In particular, we assume that the allocator wants to return a share of the available funds so as to maximize:

$$\underset{y\in [0,1]}{\text{maximize}}\;\pi^{a}\left(e_{a},X,y\right)-\frac{\alpha }{2}\;{\left(\pi^{a}\left(e_{a},X,y\right)-\pi^{i}\left(e_{i},X,y\right)\right)}^{2}$$ (1)

where *α* ≥ 0 indicates the extent to which allocators account for inequality in the final payoffs. If we take derivatives, we can derive the optimal return:

$$\begin{array}{c} y^{*}\left(X,e_{i},e_{a}\right)=\frac{2}{3}+\frac{e_{a}-e_{i}}{6X}-\frac{1}{12\alpha X} \end{array}$$ (2)

Hence, the inequality-averse allocator should return a proportion of the available funds that takes into account both the amount that he receives from the investor (*X*) and the level of endowments. More specifically, the larger the difference between the allocator and the investor’s endowment (*e*_*a*_ − *e*_*i*_) the larger the share of the available funds that an inequality-averse allocator should return. If *α* = 0 the allocator can be said to be self-interested and the optimal return will be by y* = 0. In our experiment, we shall use two different level of the endowments *e*_*k*_ ∈ {10, 40} for *k* = {*i*, *a*} that we vary across rounds. This translates into the following behavioral prediction:

**Prediction 1.** *If the allocator is inequality-averse, he will return a share of the available funds that will be affected by the level of endowments* (*e*_*i*_, *e*_*a*_), *and the larger the difference between the allocator and the investor*’*s payoffs* (*e*_*a*_ − *e*_*i*_) *the more he will return, that is,*

$$\begin{array}{c} y_{40,10}<y_{40,40}=y_{10,10}<y_{10,40} \end{array}$$

Our main interest in the paper is to test whether this relationship holds. Eventually, we can also look at the prediction of inequality-aversion for any particular distribution of endowments. As Eq (2) suggests, this will depend on the utility function that we assume for allocators (i.e., the value of *α*). A plausible assumption is to consider that allocators want to equalize payoffs *up to a point*; i.e., *α* > 0 but not sufficiently high so as to make *π*^*a*^(*e*_*a*_, *X*, *y*) equals to *π*^*i*^(*e*_*i*_, *X*, *y*) an optimal decision. In that were the case, we should expect a positive correlation between the amount sent (*X*) and the proportion returned (*y*) in the distribution (40,10). This is because for small transfers *X*, the allocator will tend to keep a high proportion of the available funds so as to restore equality. When the amount sent is sufficiently high, the allocator can restore equality by keeping a smaller proportion of the available funds, so a higher return would be expected. Similarly, a negative correlation between *X* and *y* may be expected in the (10,40) distribution. The allocator will tend to return nothing when he receives an small *X*, but he will tend to return a higher proportion of the available funds as *X* increases. One can also assume that *α* is sufficiently high so that the inequality-averse allocator would find it optimal to equalize payoffs (see [17]). If that were the case, the optimal return will be given by:

$$\begin{array}{c} y^{*}\left(X,e_{i},e_{a}\right)=\frac{2}{3}+\frac{e_{a}-e_{i}}{6X} \end{array}$$ (3)

According to Eq (3), allocators who want to restore strict equality should return 2/3 of the available funds in the absence of endowment heterogeneity (i.e., when *e*_*a*_ = *e*_*i*_). This is to keep the final distribution of payoffs equals, as it was the initial distribution of endowments. By the same token, the inequality averse allocator that wants to equalize final payoffs should return the entire available funds in (10,40). In that distribution, even if the allocator returns *y* = 1, he will be unable to equalize payoffs, despite the difference between *π*^*a*^(*e*_*a*_, *X*, *y*) and *π*^*i*^(*e*_*i*_, *X*, *y*) would be minimized. The Supporting information presents these predictions in detail. In S1 Fig, we depict the predictions of inequality aversion for each possible distribution of endowments. We show how the predictions are affected by the value of *α* > 0 in S2 Fig. This includes a discussion on the expected correlation between the amount sent and the proportion returned in each distribution of endowments. We note that some previous evidence in favor of reciprocity was based on finding a positive correlation between the amount that investors send and that allocator return, in absolute terms (e.g., [18]). However, this is not a good measure for reciprocity as already noted in [3] because the procedure “will bias the correlation statistic upwards, i.e., low amounts sent preclude some high returns” (page 131).

**Prediction 2.** *In order to restore strict equality, the inequality-averse allocator should return a share of the available funds that depend positively [negatively] on the amount received in the distribution (40,10) [(10,40)], respectively*.

We may find no evidence of inequality aversion in that allocators may return a proportion of the available funds that *i)* is consistent across distributions, and/or *ii)* does not depend on the amount received. The prediction for selfish allocators indeed establishes that allocators will return nothing to investors, regardless of the distribution of endowments or the amount received. It is also possible that allocators are reciprocal and return something positive to investors. In fact, [4] interpret any positive return from investors to allocators as evidence in favor of reciprocity. In this paper, we assume that reciprocity is fulfilled as long as the investor is not worse off than if she sent nothing; i.e., the investor should retrieve at least what she sent for the allocator to be defined as reciprocal (for a similar approach see [14], [15], [16], [17] or [18]. Because we need the investor’s payoff *π*^*i*^(*e*_*i*_, *X*, *y*) to not fall behind her initial endowment, a minimum requirement for reciprocity is that the allocator returns a proportion *y* = 1/3 of the available funds. Interestingly, this implies that the reciprocal allocator should return a fixed proportion of the available funds, regardless of the amount that he received from the investor.

## Materials and methods

We use the behavioral data in [6] to test the predictions of inequality-aversion. [6] recruited a total of 96 subjects to participate in a computerized experiment run at LINEEX (Universidad de Valencia). Their objective was to investigate the effects of punishment on the investment game, therefore two different treatments were considered: one with punishment and another one without punishment. They run these treatments using a within-subject design. At the beginning of each session, instructions (read-aloud) informed subjects that they would be assigned a role (investor or allocator) to be kept during the entire session. Each treatment was then presented to subjects as a block of 4 rounds. When playing the first block, subjects knew about the existence of a second block, but they were unaware of the specific details of the second block. Treatments were randomized so that in half of the sessions the block with (without) punishment went first.

In what follows, we describe the experimental design and the procedures for the case without punishment, which corresponds to the data we use in the current paper. We decided to focus on their treatment without punishment because any amount returned by allocators in the presence of punishment may not be explained by social preferences (e.g., inequality-aversion or a taste for reciprocity), but instead by the fear of being punished. The Wilcoxon rank-sum (Mann-Whitney) test suggests that allocators’ behavior is invariant to the order in which treatments were implement (*Z* = 1.084, p-value = 0.278).

When playing the block without punishment, subject were informed that they would play the investment game during four rounds. At the beginning of each round, subjects received an endowment that could be either 10 or 40 tokens. Subjects made a total of 4 decisions, one for each distribution of endowments (*e*_*i*_, *e*_*a*_) ∈ {(40, 10), (10, 10), (40, 40), (10, 40)}. They went though the entire sequence by the end of the experiment, but we control for the order in which distributions were played so that subjects did not play the distributions in the same order. More specifically, some subjects start playing the distribution (40,10) and then moved to (10,10), (40,40) or (10,40), whereas some other players started with (10,10) and then moved to (40,10), (40,40) or (10,40). We balance the order in which each sequence was played so as to minimize the possible influence on behavior of the order in which distributions were played. We also minimize the possibility of subjects smoothing out incentives across distributions by not informing them that they would play the four distributions of endowments. In that vein, it was common information that they would receive 10 or 40 tokens in each round and the amount that they would get in a particular round would not need to coincide with the amount received by the other player. After each round, subjects received information about their own decision and the one of their partner before being re-matched. In each block, subjects interacted using a perfect-stranger protocol; i.e., subjects never played with the same partner twice. This was implemented by considering matching groups. Each session consisted of 24 subjects, divided in 3 groups of 8 subjects. Within each group, 4 subjects were randomly assigned the role of investors and 4 subjects were assigned the role of allocators. Subjects interacted with other members of their matching-group, and subjects from different groups never interacted with each other throughout the session.

At the end of the experiment, one of the two blocks was selected for the payment. Subjects received on average 15 Euros for participating in the experiment, which lasted around 90 minutes. Supporting information contains a translated version of the original instructions. This includes screenshots of the experiment.

## Ethical approval

We understand that PLOS ONE requires an ethical approval by an institutional review board. Nevertheless, in Spain, such ethical approval is not mandatory for experimental studies that do not involve any risk or discomfort for the participants as long as anonymity is preserved (Spanish Law 15/1999 for Personal Data Protection) and participants are fully informed about the procedures of the study and give written informed consent to participate. The current experiment is in line with this regulation and further complies with the international standards of experimental economics research. The participants did not learn the identity of the other participants they interacted with and the identity of the participants cannot be inferred from the data which is entirely anonymous. Finally, the experimental protocols were approved by the LINEEX (University of Valencia), the institution hosting the experiment (see their webpage for further details about their data protection policy).

## Results

### Descriptive statistics

We summarize the data in Table 1. Panel A presents the decision of the 48 investors for each possible distribution of endowments. The data for allocators is summarized in Panel B. We note that allocators can only make a decision if they have received any transfer from investors, thus Panel B reports the behavior of those allocators who received a positive transfer from investors. This, in turn, implies that the number of observations may differ across distribution. We report the correlation between the investor’s transfer (*X*) and the allocator’s returned share (*y*) in Panel C.

**Table 1:** Summary of the data.

	*e*_*i*_ = 40		*e*_*i*_ = 10	
	(40, 10)	(40, 40)	(10, 10)	(10, 40)
**A. Investor’s behavior**				
Amount sent (*X*)	4.479	6.062	2.437	2.667
Standard Deviation	8.69	10.50	3.38	3.18
Min/Max	0/40	0/40	0/10	0/10
Proportion of zero sent	0.547	0.437	0.458	0.354
Observations	48	48	48	48
**B. Allocator’s behavior**				
Proportion returned (*y*)	0.220	0.238	0.286	0.220
Standard Deviation	0.20	0.24	0.29	0.23
95% confidence interval	(0.13, 0.31)	(0.14, 0.33)	(0.17, 0.40)	(0.13, 0.30)
Min/Max	0/0.54	0/1	0/1	0/0.66
Proportion of zero returned	0.278	0.333	0.346	0.419
Observations	22	27	26	31
**C. Correlation coefficient between *X* and *y***				
Spearman (*ρ*)	-0.030	-0.230	-0.070	-0.048
Kendall (*τ*_*b*_)	-0.039	-0.195	-0.056	-0.037

We observe in Panel A that the investor’s amount sent (*X*) increases with her endowment (*e*_*i*_), while the proportion that is sent decreases with *e*_*i*_ as already suggested in [38], [39]. We also see that investors send less when the endowment of the allocator is lower. This complements [36], where it is shown that the amount sent by investors decreases when allocators are endowed. Our findings seem to indicate that investors trust more when they are in a disadvantage position with regard to the initial distribution of endowments, what suggests a kind of discontinuity at a allocator endowment of zero. One plausible explanation for this behavior is that trust is really “altruism plus” as suggested by [10], but investors perceive that it is riskier to send money when the allocator has a low level of endowment compare with the case in which it is high. The idea in [10] is to compare investors’ giving when allocators are endowed and when they are not so as to isolate the effect of trust from the one of altruism. We note that experimental evidence on the relationship between risk attitudes and the investor’s behavior in the investment game is mixed (e.g., [27], [40]). One plausible explanation is that other factors different from risk (e.g., attitudes to betrayal) may be at stake in the investment game (see [41], [42], [43], [44]). An interesting finding along these lines is that investors are (slightly) more likely to send nothing in the (40,10) distribution, compared with the (10, 40) distribution (test of proportion: z = 1.847, p-value = 0.065, two-tailed). Investors may believe it is riskier to send money to allocators who are at a disadvantage position because they would be more likely to betray them.

As for allocators, Panel B indicates that their behavior seems to be consistent across distributions, with an average return between 22% and 28%. When doing pairwise comparisons to test if allocators’ behavior varies within distributions, we find that differences are never significant using a Wilcoxon signed-rank test (see S1 Table in Supporting information). These findings seem to contradict Prediction 1, which establishes that *y*_40,10_ < *y*_10,40_ -note that allocators return 22% of the available funds in both distributions, with the proportion of zero returned being even higher in (10, 40), where *y*_10,40_ = 1 is expected if allocators were merely concerned about equalizing final payoffs. The 95% confidence interval support this finding, with the confidence interval in (10, 40) being roughly the same as in (40, 10). This finding is also robust if we only look at the first period in which decisions are made; allocators return 0.303 in distribution (40,10) and 0.267 in distribution (10,40). The results also hold when we only look at those allocators who returned a positive amount; allocators return 0.378 in distribution (40,10) and 0.303 in distribution (10,40). In their asymmetric treatment, [4] find that allocators are less willing to return than in the symmetric one, but differences are not significant at the 5% level using a two-sided Fisher test; i.e., in their (80,40) treatment, 7 of 22 allocators return nothing (14 of 23 did it in the treatment (40,40)). In our study, allocator’s willing to pay back is roughly the same in distributions (10,10) and (40,10); where 9 of 27 and 6 of 22 allocators decided to return nothing.

Further evidence against the hypothesis that allocators want to equalize payoffs is obtained from the correlation coefficients presented in Panel C, which measure the relationship between the amount sent (*X*) and the proportion returned (*y*). These correlations are never significant (p-values > 0.202), despite one might expect a positive (and significant) correlation in the case of the distribution (40,10), and a negative (and significant) correlation in the (10, 40) distribution if allocators were concerned about equalizing payoffs (Prediction 2). These results are robust if we only consider those allocators who decided to return a positive fraction of the available funds (p-values > 0.247). We therefore conclude that the level of endowments do not affect the proportion returned and inequality-aversion receives little support from our data.

We observe that average return is around one third of the generated surplus in the symmetric distribution of endowments, as indicated by the 95% confidence interval. However, the average return is below 0.33 in the distributions (10,40) and (40, 10). When we only look at those allocators who returned a positive amount we also observe that the average proportion returned is 0.37, while the median and mode proportion returned equals 0.33 in all the distributions. We argue that reciprocity may be a plausible explanation for the allocators’ behavior (although our findings seem to be only partially consistent with this view). Self-interest seems to be an important driving force as well; in fact, the proportion of allocators returning nothing is always above 25%. We further discuss these findings in the next section, where we undertake an econometric approach. Because we identify heterogeneous behavior in the allocators’ choices, we also rely on an individual analysis to assess the predictive power of inequality-aversion.

### Econometric results

Table 2 reports the maximum-likelihood estimates of three different random effects specifications that aim at explaining the allocators’ behavior and control for unobserved individual heterogeneity. The Breusch and Pagan Lagrangian multiplier test supports the random-effects specification, but our findings are also robust to other specifications (e.g., if we cluster at the individual level or consider subject fixed-effects. The dependent variable in all the models refers to the proportion of the available funds that the allocator returns, *y* ∈ [0, 1]. In Model (1), the set of independent variables include the amount received (*X*), the period in which the decision is made (Period), and the treatment conditions, where the dummy variable $I_{e_{i},e_{a}}$ that takes the value 1 if the distribution of endowment is (*e*_*i*_, *e*_*a*_). Model (2) considers the same set of independent variables with the one exception of the amount received (*X*), which is now replaced by the proportion of the endowment the investor sent (*X*/*e*_*i*_). Model (3) again considers the amount received but controls for differences in the endowments by including a dummy variable $I_{HIGHe_{i}}$ that takes the value 1 when the investor is endowed with 40 tokens. This dummy is interacted with the amount received by the allocator to see if there is any difference in behavior depending on whether the allocator receives the transfer from an investor with a high or a low level of endowment. This is intended to capture the importance of intention-based models and the possibility that allocators value differently the amount received by a rich or poor investor. For each model, we consider two diffregressions: model (a) that does not control for demographics, and model (b) which does include gender, age and attitudinal trust as controls (see, among others, [45], [46], [47], or [49] for evidence on gender differences, and [48] or [50] for the predictive power of attitudinal trust). The reported standard errors (in brackets) take into account matching group clustering, as suggested in [51].

**Table 2:** Random-effect estimates for the allocator’s behavior.

	No controls			Controls		
	Model (1a)	Model (2a)	Model (3a)	Model (1b)	Model (2b)	Model (3b)
Constant	0.351*** (0.058)	0.388*** (0.072)	0.372*** (0.065)	0.167 (0.189)	0.223 (0.168)	0.210 (0.158)
Amount received (*X*)	-0.002 (0.003)		-0.0118 (0.008)	-0.003 (0.002)		-0.010 (0.008)
Proportion received (*X*/*e*_*i*_)		-0.096 (0.076)			-0.090 (0.062)	
Period	-0.032* (0.017)	-0.034** (0.017)	-0.034** (0.017)	-0.032* (0.017)	-0.034** (0.017)	-0.033** (0.016)
*I*_10,40_	-0.043 (0.065)	-0.046 (0.066)		-0.042 (0.080)	-0.045 (0.080)	
*I*_40,10_	-0.058 (0.063)	-0.090 (0.069)		-0.059 (0.056)	-0.090 (0.065)	
*I*_40,40_	0.002 (0.068)	-0.033 (0.066)		0.008 (0.050)	-0.0261 (0.057)	
$I_{HIGHe_{i}}$			-0.054 (0.069)			-0.042 (0.065)
Amount received x $I_{HIGHe_{i}}$			0.010 (0.008)			0.0085 (0.008)
Gender (= 1 if female)				0.054 (0.080)	0.050 (0.080)	0.041 (0.077)
Age				0.007 (0.010)	0.006 (0.010)	0.006 (0.009)
Trust (GSS)				-0.017 (0.097)	-0.017 (0.095)	-0.012 (0.098)
*σ*_*u*_	0.147	0.137	0.149	0.154	0.146	0.158
*σ*_*e*_	0.194	0.194	0.197	0.194	0.194	0.197
*ρ*	0.367	0.333	0.362	0.387	0.364	0.391
R-squared	0.042	0.060	0.050	0.069	0.076	0.068
Wald test	17.15***	12.65**	12.46**	39.33***	15.53**	40.45**
Observations	106	106	106	106	106	106

Note: Standard errors in parentheses are clustered by groups. The independent variable Trust (GSS) correspond to the answer in the attitudinal survey question from the General Social Survey: “Generally speaking, would you say that most people can be trusted or that you cannot be careful in dealing with people?” (= 1 if most people can be trusted). Significance at the **** p<0.01, ** p<0.05, * p<0.1 level.

Overall, our results are in line with our previous discussion in that inequality aversion cannot be supported by our data. Inequality aversion predicts a higher (smaller) return in *I*_10,40_ (*I*_40,10_), compared with the baseline distribution. However, we find that our estimates for the treatment variables *I*_10,40_ and *I*_40,10_ are not statistically different from zero. In fact, the estimates for the treatment conditions *I*_10,40_ and *I*_40,10_ are statistically indistinguishable from each other at any common significance level (p-values > 0.567, without controls; p-values > 0.519, with controls). To further see that the investor’s endowment does not affect the allocator’s decision, we can see the estimate for the dummy variable $I_{HIGHe_{i}}$ in Model (3). This is not statistically different from zero, despite one would expect a negative effect of *e*_*i*_ = 40 if the allocator were inequality averse.

Recall that we can also test inequality aversion within each particular distribution, once we assume a particular utility function (i.e., a value of *α*). One other plausible assumption is to consider that allocators want to restore strict equality (see [17]). If that were the case, allocators would keep all what is generated in the distribution (10,40). Using models (1) and (2), we reject that hypothesis at any common significance level (with and without controls, p-values <0.001). Along similar lines, we reject the null hypothesis that the constant equals 2/3 and the estimate for the amount received equals 0 simultaneously (*H*_0_: *β*_*Constant*_ = 2/3, *β*_*received*(*X*)_ = 0) in both distributions (10,10) and (40,40) using Models (1) and (2) (without controls, p-values <0.001, with controls, p-values <0.05). Finally inequality aversion predicts a positive relationship between the amount sent and the proportion returned in the (40,10) distribution. Using model (3), we cannot reject the null hypothesis that $H_{0}:\beta_{received(X)}+\beta_{received(X)I_{HIGHe_{i}}}=0$) at any common significance level (p-value = 0.486). This, in turn, indicates that the amount received does not affect the allocators’ decision in the (40,10) distribution, in line with the correlation coefficients in Table 1. Thus, inequality aversion seems to receive little support using our econometric analysis.

Our estimates in Table 2 indicate also that the amount or the proportion received do not affect the allocators’ return. This is an interesting finding that is sometimes interpreted as evidence against reciprocity. In our setting, we ask allocators to return at least one third of the available funds to be classified as reciprocal. We find evidence of reciprocity in the baseline distribution. In this distribution, the null hypothesis that the estimate for the constant equals 1/3 and the estimate for the amount received equals 0 simultaneously (*H*_0_: *β*_*Constant*_ = 1/3, *β*_*received*(*X*)_ = 0) cannot be rejected at any common significance level (without controls, p-values > 0.456; with controls, p-values > 0.395). The same holds when we test for reciprocal behavior in the rest of the distributions. The p-values are always larger than 0.187 except when we test for reciprocity in the distribution (10,40) in Models (1a) and (2a) (p-values = 0.095 and 0.061). We therefore conclude that reciprocity receives some support from our data, but cannot fully explain the allocators’ behavior. As a matter of fact, there is a substantial proportion of selfish allocators. The estimated constants in Table 2 are always significant without controls (p-values < 0.0001) but become insignificant with controls.

Finally, our results in Table 2 indicate that allocators tend to return a smaller proportion of the available funds with the period, which is a common pattern in other experiments in which there is repetition (e.g., public good games).

### Individual analysis

In this section, we attempt to shed some light into the individual heterogeneity we have identified, thus we consider an analysis at the individual level. First, we consider the best possible scenario in which inequality aversion could be used to explain the data in that we do not impose any value of *α* but simply expect for any inequality-averse allocator to return (less) more when her endowment (the endowment of the investor) increases, *ceteris paribus*. This is the weakest definition of inequality-aversion one may consider, thus we will require that most of the choices are in line with this prediction to support the idea of inequality aversion. Our second approach relies on a strict definition of inequality aversion and assumes that allocators may want to restore strict equality. We then classify choices and subjects as selfish, inequality-averse or reciprocal, depending on the extent to which their choices are consistent with each criteria.

#### Weak definition of inequality aversion

For any two distribution of endowments in which inequality aversion posit a clear-cut prediction, we report in Table 3 the number of allocators that behaved in the expected direction (the frequency appears in brackets). For example, when we compare (10,10) and (10,40), inequality aversion predicts that allocators will return less in the former distribution. Table 3 shows that 6 out of 17 allocators (35.39%) behaved according to that pattern and returned more in (10, 10) than in (10, 40). Similarly, 4 out of 14 allocators (28.57%) returned more in (10, 10) than (40, 10), as predicted by inequality-aversion.

**Table 3:** Frequency of choices that are consistent with the predictions of inequality aversion.

	*N*	Prediction	Observed
(10,10) vs (10,40)	17	*y*_10,10_ < *y*_10,40_	6 (35.29%)
(10,10) vs (40,10)	14	*y*_40,10_ < *y*_10,10_	4 (28.57%)
(10,40) vs (40,40)	17	*y*_40,40_ < *y*_10,40_	6 (35.29%)
(40,10) vs (40,40)	13	*y*_40,10_ < *y*_40,40_	8 (61.54%)
(10,40) vs (40,10)	30	*y*_40,10_ < *y*_10,40_	11 (36.67%)
Overall	91		35 (38.46%)

Overall, there are 91 situations in which the idea of inequality aversion predicts a change in the allocators’ behavior when we vary the level of endowments. We observe that less than 40% of the times, allocators behaved in the predicted direction (i.e., more than 60% of choices were inconsistent with inequality-aversion). While this is a substantial proportion of choices, the binomial test rejects the hypothesis that majority of the choices are in the direction predicted by inequality aversion (p-value < 0.018, one-sided test).

#### Strong definition of inequality aversion

**Fig 1:**
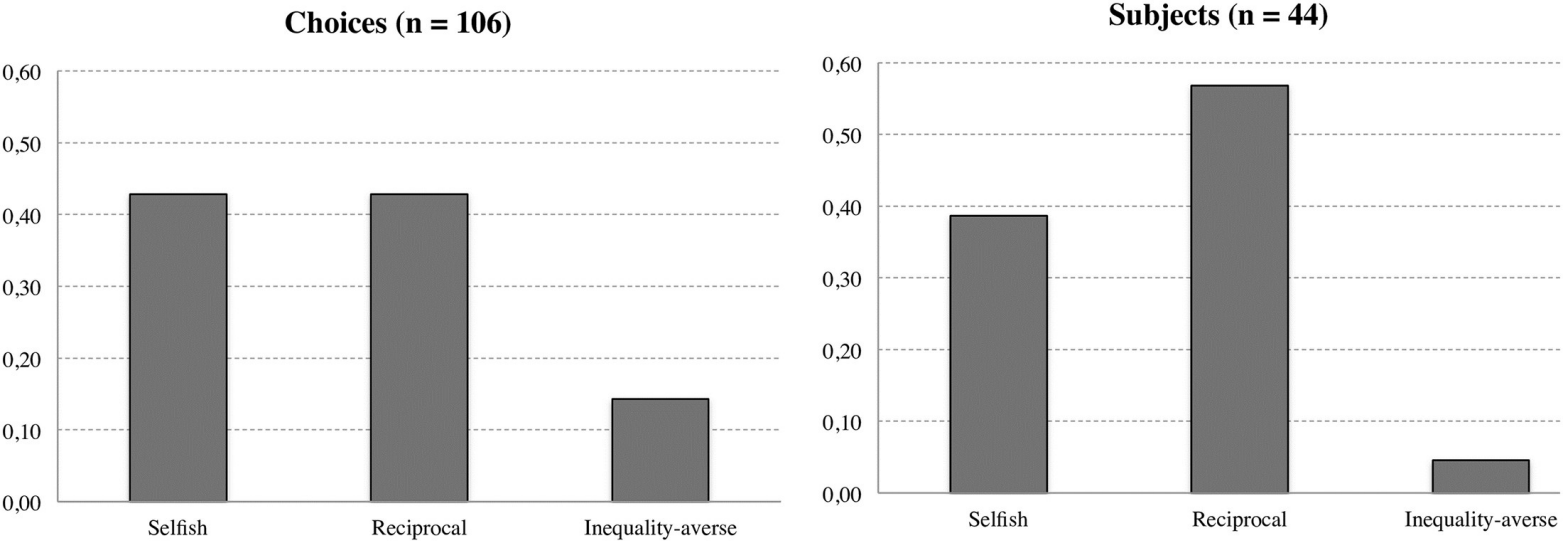
Classification of choices and subjects. We consider three behavioral motives to explain the allocators’ behavior. Selfishness requires returning nothing, reciprocity requires returning what has been received and inequality aversion requires restoring strict inequality. Choices and subjects are classified using the mean square error criteria.

Next, we use the assumption in [17] that inequality-averse subjects want to restore strict equality to investigate heterogeneity across subjects. We consider that there are three different behavioral patters to explain our data. Allocators can keep the available funds and transfer y* = 0 if selfish. Allocators can also return what they have received from investors y* = 1/3 if reciprocal. Finally, allocators can be inequality-averse and return a proportion of the available funds that depend on the level of endowments and what they have received from investors, y*(*X*, *e*_*i*_, *e*_*a*_), as indicated in Eq (3). In Fig 1, we classify choices using the minimum distance to each of the three behavioral motives. We rely on the mean square error criteria to classify subjects as selfish, reciprocal or inequality-averse. When a choice can be explained by two principles, we select one of them at random. For instance, a return of y = 0.165 in the distribution (10,40) can be explained by reciprocity and also by selfish behavior because the distance to both principles is the same. We select one of the two principles randomly, and assume that the choice is guided by either reciprocity or selfish behavior. The proportion of overlaps is very small, about 6 percent of all decisions.

A first thing to notice is that the large majority of choices (more than 90%) are either purely selfish or can be classified using our definition of reciprocity, both behavioral motives being equally important in explaining allocators’ choices. Less than 10% of choices are classified according to our definition of inequality aversion. At the subject level, the results also seem to be conclusive. Less than 5% of allocators make choices that are better explained by inequality than by selfish or reciprocal behavior. Overall, these findings are in line with our results suggesting that inequality aversion does not greatly matter if we want to explain allocators’ behavior.

## Discussion

This paper has attempted to investigate whether allocators in the investment game are motivated by inequality-aversion and thus return more (less) to investors who were endowed with a smaller (higher) endowment. We used the behavioral data in [6] to test this behavioral motive using a within-subject design where subjects play the investment game repeatedly with different level of endowments that vary across rounds.

Our results lend little support in favor of the predictions of inequality aversion in that allocators do not tend to return a larger (smaller) fraction of the available funds if their endowment is larger (smaller) than that of the investors. Instead, we find that allocators have a tendency to reciprocate investors’ behavior by sending a share of the available funds that is consistent across distributions. There is also a non-negligible proportion of selfish allocators in our data.

The assumption that subjects are willing to forego monetary payoffs to reduce inequality has served to explain and predict a large swath of observed behavior in laboratory experiments. Although we agree that inequality aversion may have some predictive power in different settings, our results provide little evidence in that direction. One plausible explanation to rationalize our findings is to consider that allocators do not held themselves responsible for the initial distribution of endowments (which is exogenously determined) and thus simply focus on what is available to be distributed when making their choices. This, in turn, implies that allocators will focus on equalizing the distribution of the available funds rather than taking care of equalizing final payoffs. This is someone related to the accountability principle in the theory of justice ([52], [53], [54], [55], which posits that subjects may not feel responsible for factors beyond their control. The within-subject design may have contributed to our findings as well. Subjects play the investment game in four different rounds and do not know ex-ante how their endowments will change across rounds (nor they know the way in which the endowment of their counterpart will change across rounds). In that vein, allocators may want to appear as “consistent” by always returning the same share, especially because all rounds can be paid at the end of the experiment. Further, our experimental setting is such that subjects interact with different partners across rounds and this may generate moral wiggle room ([56]). In particular, allocators may rely on the presence of other allocators to reduce the inequality; i.e., they may want to believe that other allocators will compensate investors with a low endowment. Allocators may also want to believe that investors with low endowments will receive a high endowment in future rounds, which may prevent them from restoring equality. The moral wiggle room has been recently studied in the investment game by [57]. The authors study the effect of “plausible deniability” by considering a setting with time pressure in which the allocator has a time limit to make a decision; otherwise, the computer will choose whether or not implementing an equal distribution. Unlike our setting, they do not consider any difference in the level of endowments nor they allow for repeated decisions. Finally, it is also possible that our sample composition matters for the results. Using experimental data from dictator and ultimatum games, the structural estimates in [20] suggest that young and highly educated subjects have lower aversion for inequity than other subjects. As we conducted our experiments using university students, it is likely that our subjects display less aversion to inequality than others in the population.

Our study, however, is the not the first one in which inequality aversion is not supported by the data (e.g., [22], [23], [24], [26]). In fact, our evidence is in line with the low predictive power of inequality aversion at the individual level in [58]. While we argue that reciprocity is a possible explanation to reconcile with our data, it is also true that a substantial proportion of allocators are selfish, and there are other possible motives (such as altruism) that could potentially explain our findings. Our design does not allow -and it was not intended to allow- for discrimination between different explanations when allocators return a positive amount to investors ([10], [11]). Using a within-subject design, our aim was to study the predictive power of inequality-aversion, which predicts a different effect of the level of endowments on the allocator’s behavior. In that regard, it may be worth investigating how different behavioral motives can explain choices, especially when the context changes ([58]). The between and within-subjects comparison might also relevant to test inequality aversion, thus we believe this is also a good avenue for future research.

Overall, we share the view advanced in [4] or [22] that further research is needed for the particular case of the investment game to investigate subjects’ behavior when different motives underlying decision making are in conflict. We consider our paper to be a contribution on these lines and hope our results will provide the impetus to further research in this area.

## Supporting information

S1 Fig Behavior in the investment game under reciprocity and inequality aversion. (TIF)

S2 Fig Optimal return (*y*) under inequality aversion for different values of alpha. (TIF)

S1 Table Non-parametric analysis to test the allocator’s behavior across distributions. (DOCX)

S1 File Supporting information. (DOCX)
