# Supplementary figures and images for: No evidence of inequality aversion in the investment game
Source: PLoS One. 2018 Oct 23;13(10):e0204392. doi: 10.1371/journal.pone.0204392 (PMC6198942; doi:10.1371/journal.pone.0204392)

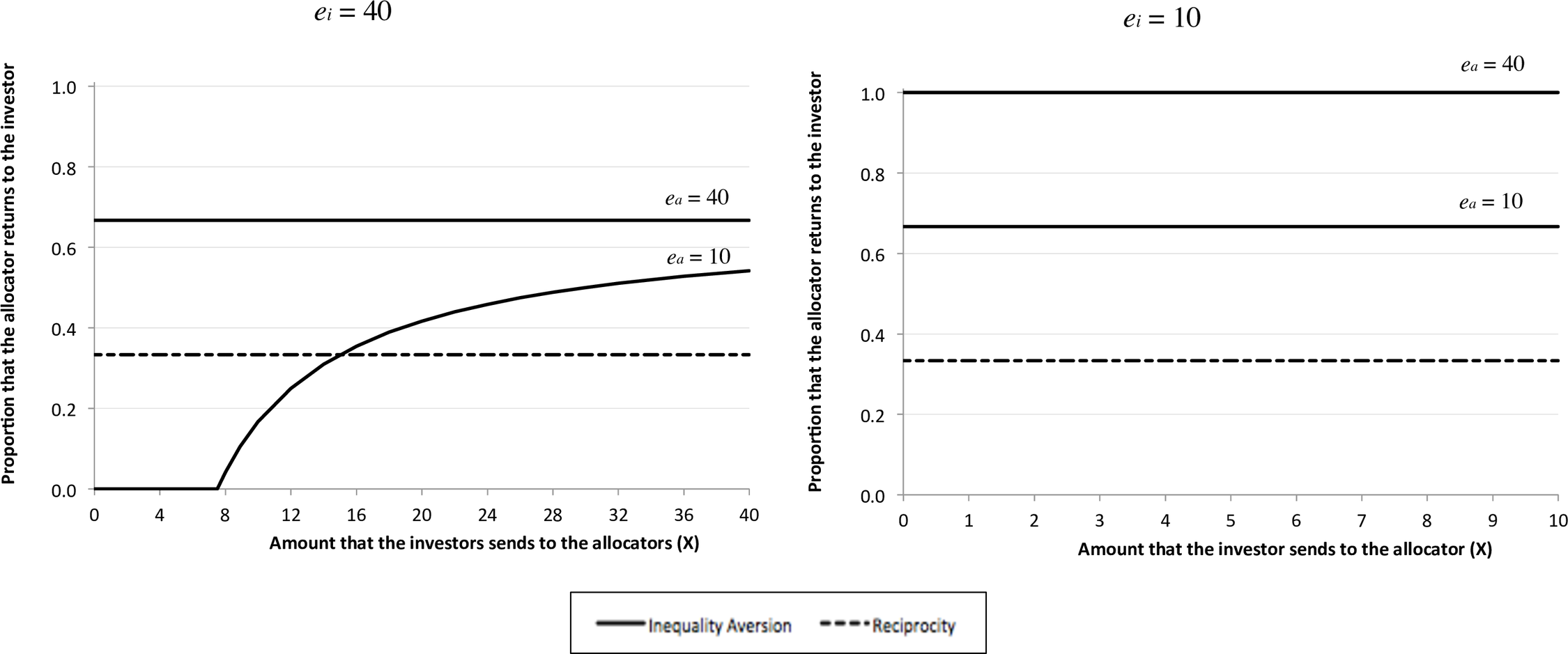

Supplement: S1 Fig — (TIF) [file pone.0204392.s001.tif]

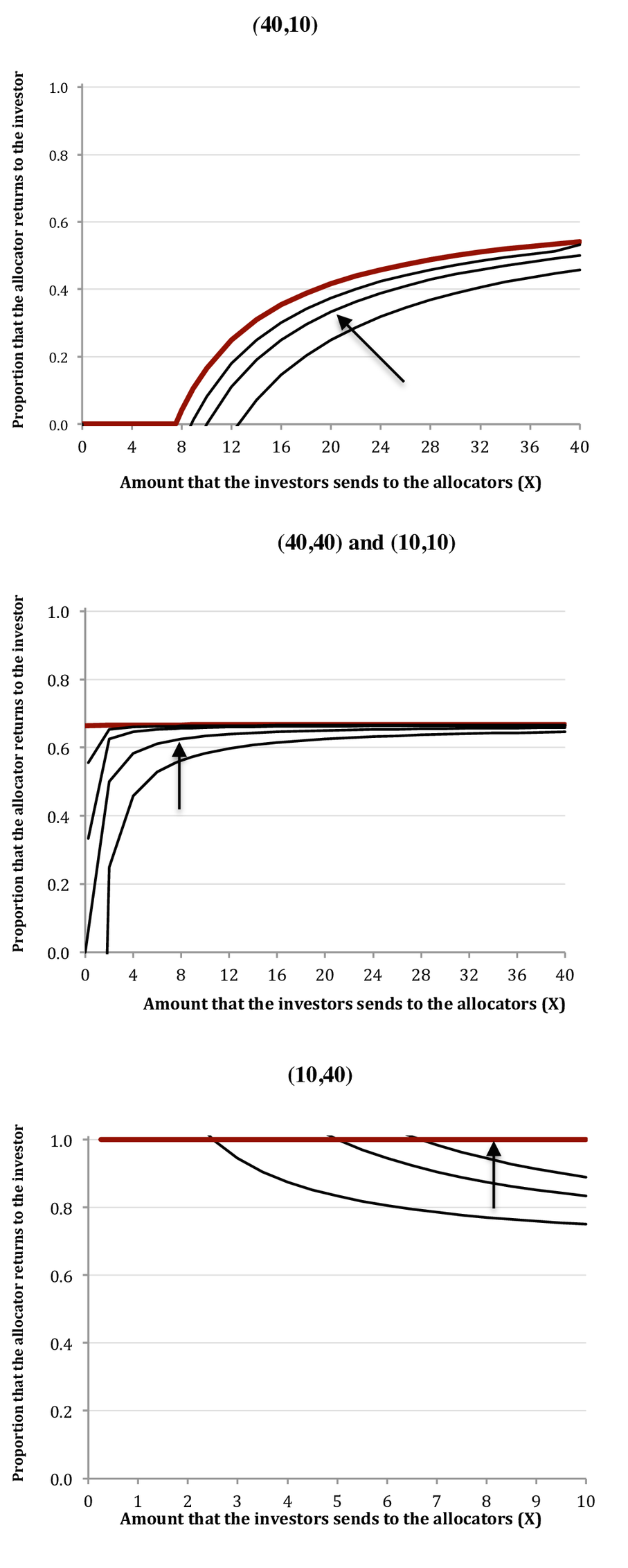

Supplement: S2 Fig — (TIF) [file pone.0204392.s002.tif]
