# Supplementary material for: No evidence of inequality aversion in the investment game
Source: PLoS One. 2018 Oct 23;13(10):e0204392. doi: 10.1371/journal.pone.0204392 (PMC6198942; doi:10.1371/journal.pone.0204392)
Supplement: S1 Table — (DOCX) [file pone.0204392.s003.docx]

**S1 Table.** Non-parametric analysis: comparison of proportion returned across distribution of endowments

|  | $\boldsymbol{y}_{\boldsymbol{40,40}}$ | $\boldsymbol{y}_{\boldsymbol{10,10}}$ | $\boldsymbol{y}_{\boldsymbol{10,40}}$ |
| --- | --- | --- | --- |
| $\boldsymbol{y}_{\boldsymbol{40,10}}$ | -1.645  (0.100) | -0.129  (0.897) | 0.736  (0.462) |
| $\boldsymbol{y}_{\boldsymbol{40,40}}$ |  | 1.607  (0.108) | 0.523  (0.601) |
| $\boldsymbol{y}_{\boldsymbol{10,10}}$ |  |  | -0.629  (0.529) |
