# Supplementary material for: No evidence of inequality aversion in the investment game
Source: PLoS One. 2018 Oct 23;13(10):e0204392. doi: 10.1371/journal.pone.0204392 (PMC6198942; doi:10.1371/journal.pone.0204392)
Supplement: S1 File — (DOCX) [file pone.0204392.s004.docx]

**Supplementary Information**

**No evidence of inequality aversion in the investment game**

**Ismael Rodriguez-Lara**

Middlesex University London

**Theoretical predictions**

Recall that the investor and the allocator’s payoffs in the investment game in Berg et al. (1995) are given by:

(1) $\pi^{i}$($e_{i},$ X, y) :=$e_{i}$+ X (3y – 1) ≥ 0

(2) $\pi^{a}$($e_{a},$ X, y) := $e_{a}$+ 3X (1 – y) ≥ 0

where $e_{i}$≥ 0 and $e_{a}$ ≥ 0 denote the investor (subject *i*) and the allocator’s (subject *a*) endowments, respectively, the value of X in [0,$e_{i}$] corresponds to the amount that the investor sends to the allocator, and *y* in [0,1] stands for the percentage of the available funds that the allocator decides to return.

If we consider the assumption in Coleman (1990) and Ciriolo (2007), a minimum requirement for reciprocity is that the allocator returns at least one third of the available funds ($y=1/3$), or otherwise the investor would be worse off than if she have sent nothing. As for inequality aversion, the allocator may want to equalize the final payoffs, so that the initial distribution of endowments may play a role in his decision (Ciriolo 2007, Xiao and Bicchieri 2010). To build upon this possibility, we can follow Ciriolo (2007) and impose equality of payoffs, $\pi^{i}$($e_{i},$X,y) = $\pi^{a}$($e_{a},$X,y). If we solve for the allocator’s decision, we obtain the result that the allocator who wants to equalize payoffs should return a fraction of the available funds that takes into account both the amount that he receives from the investor and the level of endowments. Thus, we can define an allocator as *inequality averse if he returns a proportion y ∈ [0,1] of the available funds that satisfies:* $y=\frac{e_{a}-e_{i}}{6X}+\frac{2}{3}$ .

In the absence of endowment heterogeneity (i.e., when $e_{i}=e_{a}$) the inequality-averse allocator will return 2/3 of the available funds. This is to keep the final distribution of payoffs equal, as it was the initial distribution of endowments. When the investor’s endowment is larger than the allocator’s one (i.e., $e_{i}>e_{a}$), a higher X implies that the inequality-averse allocator will return a larger share of the available funds. This is because for small values of X, the allocator will tend to keep the available funds and return nothing so as to reduce payoff inequalities.

Next, we use an example to illustrate the predictions of the reciprocal and the inequality-averse allocator in each possible distribution of endowments. Recall that we use two level of endowments in our experiment, $e_{k}\in$ {10,40} for $k=i,a$. Along the horizontal axis of S1 Fig. we plot the investor’s decision X $\in$ [0,$e_{i}$]. We use the vertical axis to represent the allocator’s decision, y $\in$ [0,$1$]. Fig. 1a depicts the case of $e_{i}=$ 40 tokens. The reciprocal allocator should return y = 1/3, regardless of his endowment or the amount he received from the investor. If he is inequality averse, and endowments coincide (i.e., $e_{a}=40$), the amount received will play no role in the allocator’s decision either, and y = 2/3. However, when the initial distribution of endowments is in favor of the investor (i.e., $e_{a}=10$), inequality aversion predicts that the allocator will return nothing if he receives less than X = 7.5 tokens. As the allocator receives more money, he will return a larger fraction of the available funds, and will end up transferring y ≅ 0.54 if he receives 40 tokens (i.e., out of the 120 tokens generated with the investor’s decision, the allocator keeps 55 tokens to be added to his initial endowment of 10 tokens; and returns 65 tokens).

The right-hand side panel of Fig 1A plots the case in which $e_{i}=10$. Again, the reciprocal allocator should return y=1/3 and the inequality averse allocator y = 2/3 if endowments are equal ($e_{a}=10$). Now, the reciprocal allocator should return the entire available funds (y = 1) when the initial distribution of endowments is in his favor ($e_{a}=40$) so as to minimize payoff differences.^[[1]](#footnote-1)^

**S1 Fig.** Behavior in the investment game under reciprocity and inequality aversion

Our aim is not to study whether reciprocity or inequality aversion can explain behavior within particular distribution of endowments, but the extent to which both two behavioral motives can be reconciled with our data when we change the distribution of endowments. In that regard, reciprocity predicts that changing the endowments will not affect the allocator’s choices, whereas inequality aversion predicts higher returns in (10,40) compared with (40,10).

One aspect that is worth mentioning is that our definition of reciprocity implies not only that the return will be consistent across treatments but also that allocators will return one third of the available funds regardless of the distribution of endowments. Importantly, our models of reciprocity and inequality aversion predict no relationship between the allocator’s return (y) and the amount received from investors (X), except in the distribution (40,10), where a positive relationship between X and y is expected if allocators are inequality averse (especially when the allocator receives more than 7.5 tokens) (see S1 Fig.). This is because we are considering the (extreme) assumption that allocators want to restore equality of payoffs if they are inequality averse; i.e., $\pi^{i}$($e_{i},$X,y) = $\pi^{a}$($e_{a},$X,y) should be satisfied for the allocator to be considered as inequality averse. Arguably, we can assume that allocators dislike payoff differences but not as much as to equalize payoffs. We can then consider that allocators, if inequality-averse, choose to return a proportion of the available funds so as to maximize:

Max*_{y}_* $\pi^{a}$($e_{a},$ X, y) – $\frac{\alpha}{2}\left( \pi^{a}(e_{a}, X, y) - \pi^{i}(e_{i}, X, y) \right)^{2}$

In this case, the optimal decision for the allocator if inequality averse will depend not only on the received amount (X) and the level of endowments, but also on the value of $\alpha$, as follows:

$$y=\frac{e_{a}-e_{i}}{6X} + \frac{2}{3} -\frac{1}{12 \alpha X}$$

S2 Fig. shows the allocator’s decision depending on the particular value of $\alpha$ for each possible distribution of endowments separately.^[[2]](#footnote-2)^ An arrow indicates how the predictions change when the allocator is more adverse to inequality (i.e., when we increase the value of $\alpha$). We observe that optimal choices tend to the ones represented in S1 Fig. when the value of $\alpha$ is sufficiently large so that the allocator wants to restore strict equality (red lines).

**S2 Fig.** Optimal return (*y*) under inequality aversion for different values of $\alpha$

We observe in S2 Fig that inequality aversion predicts a positive correlation between the amount sent and the proportion of the available surplus returned in the distribution (40, 10). It is also the case in the absence of endowment heterogeneity (i.e., when $e_{i}=e_{a}$), although choices collapse to an optimal return of 2/3 of the available funds when we increase $\alpha$ (i.e., when the allocator is sufficiently inequality averse then he will return 2/3, regardless of what he has received). Similarly, inequality aversion predicts a positive correlation between amount sent and proportion returned in (10, 40), except for the case in which the inequality-averse allocator wants to restore strict equality. In that case, he should return all what is generated, therefore no correlation between X and y will be expected.

**S1 Table. Non-parametric analysis to test the allocator’s behavior across distributions.**

We perform a Wilcoxon matched-pairs signed-ranks test for the null hypothesis that the allocator’s behavior is the same in any two distributions.^[[3]](#footnote-3)^ We report the value of the statistic and the p-value (in brackets), which are not corrected for multiple testing (i.e., we should expect higher p-values if we undertake the Bonferroni correction).

Recall that our hypothesis for inequality aversion is such that $y_{40,10}<y_{40,40}=y_{10,10}<y_{10,40}$. We can therefore conclude that inequality-aversion is not supported by our data using a non-parametric approach.

1. Note that for any amount X $\in$ [0,10] that the allocator receives, inequality aversion predicts a return y > 1. This is not possible in our framework, as the allocator cannot transfer part of his own endowment to the investor. In the model of Ciriolo (2007), allocators are allowed to do so, and equality of payoffs is considered as a sufficient condition for reciprocity. [↑](#footnote-ref-1)
2. We do not show the predictions in (10, 10) because these are similar to the ones in (40, 40) as in both cases$e_{a}=e_{i}$. [↑](#footnote-ref-2)
3. Because allocators can only return money if they have received it, when we compare two distributions we only consider those allocators who received money in both of them. This is why the number of observations varies across comparisons. The minimum sample size (13 observations) is when we compare distributions (40,10) and (10,40), and the maximum (30 observations) when we compare (40,10) and (10,40). [↑](#footnote-ref-3)
